# Regulatory science promotes the translation of transcatheter tricuspid valve repair/replacement devices

**DOI:** 10.1093/rb/rbae084

**Published:** 2024-07-18

**Authors:** Maobo Cheng, Yun Xu, Wei Liu, Lanlan Mu, Xiaoqi Lian, Guobiao Gao, Lei Sun

**Affiliations:** Center for Medical Device Evaluation, National Medical Products Administration, Beijing 100081, China; Center for Medical Device Evaluation, National Medical Products Administration, Beijing 100081, China; Center for Medical Device Evaluation, National Medical Products Administration, Beijing 100081, China; Center for Medical Device Evaluation, National Medical Products Administration, Beijing 100081, China; Guangdong-Hong Kong-Macao Greater Bay Area, Center for Medical Device Evaluation and Inspection of NMPA, Shenzhen 518045, China; Center for Medical Device Evaluation, National Medical Products Administration, Beijing 100081, China; Center for Medical Device Evaluation, National Medical Products Administration, Beijing 100081, China

**Keywords:** transcatheter interventional products, transcatheter tricuspid valve repair devices, transcatheter tricuspid valve replacement devices, regulatory science, translation

## Abstract

For patients with symptomatic and severe tricuspid regurgitation but inoperable with open surgery, transcatheter tricuspid valve intervention (TTVI) is a procedure of great clinical value. TTVI products include repair and replacement devices. TTVI products are one of the hotspots of investigation now, with different innovative biomaterials and structural designs in trials to satisfy divergent indications and reduce complications. With the emerging biomaterials, the technical difficulty of structural design will be greatly reduced, spurring further product innovation and development. The innovativeness and complexity of TTVI products have brought challenges to academia, industry, and regulatory agencies. Regulatory science provides a bridge to address these difficulties and challenges. This perspective article introduces the latest development of the TTVI products. With traditional methods, regulatory agencies face challenges in evaluating the safety and efficacy of TTVr/TTVR devices given the uncertainty of clinical use and the diversity of innovative structural design. This perspective article analyzes the regulatory challenges and discusses regulatory science that can be developed to assess the safety, efficacy, quality and performance of such products: including new approaches for innovative devices, pre-review path, computer modeling and simulation, accelerated wear testing methods for transcatheter heart valves and evidence-based research. This article reveals for the first time how to apply regulatory science systematically to TTVI products, which is of great relevance to their development and translation.

## Graphical abstract

**Figure:**
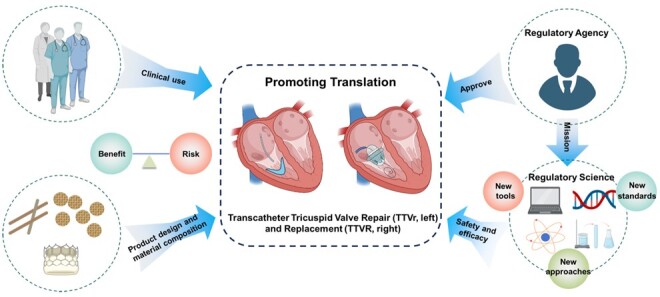

## Introduction

More than 70 million people worldwide suffer from clinically significant tricuspid regurgitation (TR) [1, 2]. As the most common form of TR, secondary or functional TR results from cardiomyopathy, left ventricular valvular disease, pulmonary disease or the dilation of the tricuspid valve (TV) annulus due to the right ventricle or right atrium remodeling [3–5]. Surgical valve repair or replacement is the gold standard for the treatment of TR. Medical guidelines that recommend the surgical treatment of TR are increasing by the year.

**Figure 1.**
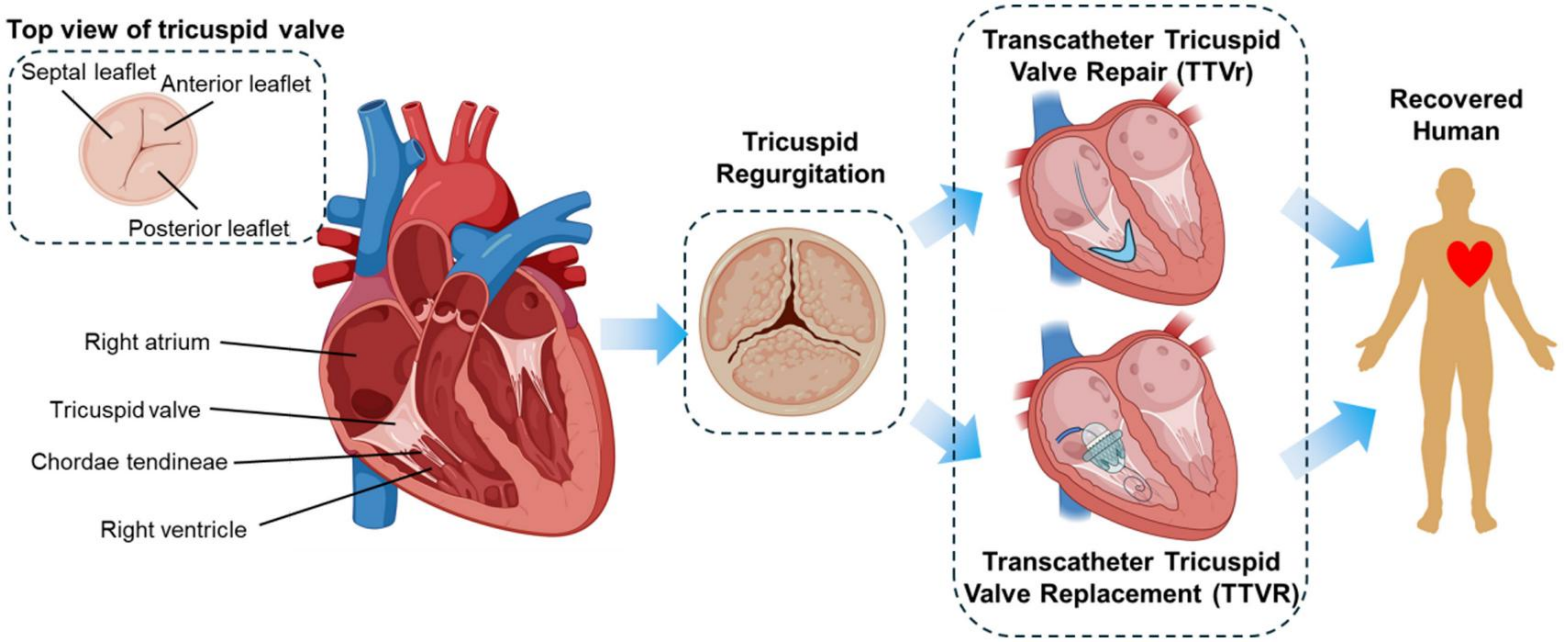
The normal human TV is divided into the septal leaflet, anterior–superior leaflet, and inferior leaflet. TR is the most common TV disease. TTVI products, namely TTVr and TTVR devices, are clinically significant for the treatment of symptomatic, severe, and inoperable TV regurgitation but unsuitable for open surgery.

Surgical risks are high in patients with functional TR who have a left ventricular ejection fraction <40%, severely impaired right ventricular function and pulmonary hypertension [3]. Transcatheter tricuspid valve intervention (TTVI) shows promise in improving quality of life and reducing mortality in patients. The 2021 ESC/EACTS guidelines for the management of valvular heart disease recommends TTVI intervention for the first time and emphasizes the necessity of early TV intervention [6]. For patients with symptomatic, severe and inoperable TR, treatment with transcatheter interventional procedures at experienced medical centers is recommended. Transcatheter interventional procedures typically include transcatheter tricuspid valve repair (TTVr) or replacement (TTVR) (Figure 1).

TTVI products are one of the hotspots of investigation now, with different innovative biomaterials and structural designs in trials to satisfy divergent indications and reduce complications. With the emerging biomaterials, the technical difficulty of structural design will be greatly reduced, spurring further product innovation and development. The innovation and complexity of TTVI products have brought difficulties and challenges to academia, industry and regulatory agencies, which might be overcome by rigorous regulatory science, ‘the science of developing new tools, standards, and approaches to assess the safety, efficacy, quality, and performance of regulated products’ [7–9].

This article aims to reveal how regulatory science promotes the research, development, translation and fulfillment of regulatory needs for TTVI products. First, the state of development of TTVI devices is introduced. Then, the regulatory challenges are analyzed. Finally, the regulatory science of TTVI products is discussed.

## Development of TTVr/TTVR devices

There are currently many TTVI products under development. This section introduces mechanisms of action, material composition and representative products.

### TTVr

**Figure 2.**
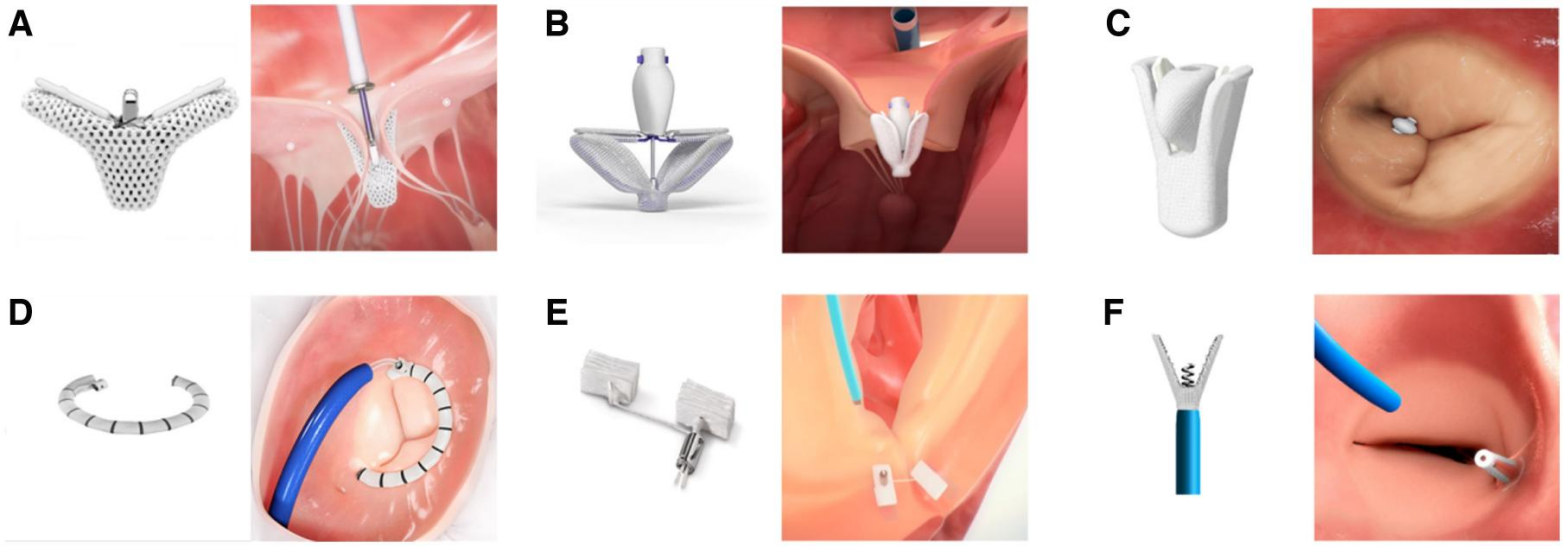
Representative TTVr devices. (**A**) TriClip G4, (**B**) pascal, and (**C**) DragonFly-T are for TEER. The mechanism of action is to form a ‘bivalve’ structure by clipping two TV leaflets together to reduce the severity of TR. (**D**) Cardioband, (**E**) trialign, and (**F**) K-clip are transcatheter tricuspid annuloplasty systems that aim to reduce TV annular diameter and the severity of TR. Images were provided by the manufacturers.

TTVr refers to surgical intervention of suture-based annuloplasty (such as Kay’s and DeVega procedures), and TV annuloplasty, which involves transcatheter edge-to-edge repair (TEER) and transcatheter tricuspid annuloplasty [3]. The mechanism of action of TEER is to use valve clips to attach the anterior and/or posterior leaflet to the septal leaflet. Clipping two tricuspid leaflets helps form a ‘bivalve’ coaptation structure for repair [3]. The mechanism of action for annulus repair is to treat regurgitation by reshaping, strengthening or tightening the annulus around the heart valve, optimizing the junction area, maintaining the mobility of the valve leaflets and preventing further expansion of the annulus [3]. A TTVr device usually consists of components made of metallic materials (such as stainless steel, nickel-titanium alloy and cobalt-chromium alloy), and polymer materials (such as polyesters) [4, 10, 11]. Currently, there are CE-marking TTVr devices, such as TriClip and Pascal for TEER, and Cardioband for transcatheter tricuspid annuloplasty. Among them, TriClip was backed unanimously by the FDA Advisory Panel. The representative TTVr devices are shown in Figure 2.

### TTVR

**Figure 3.**
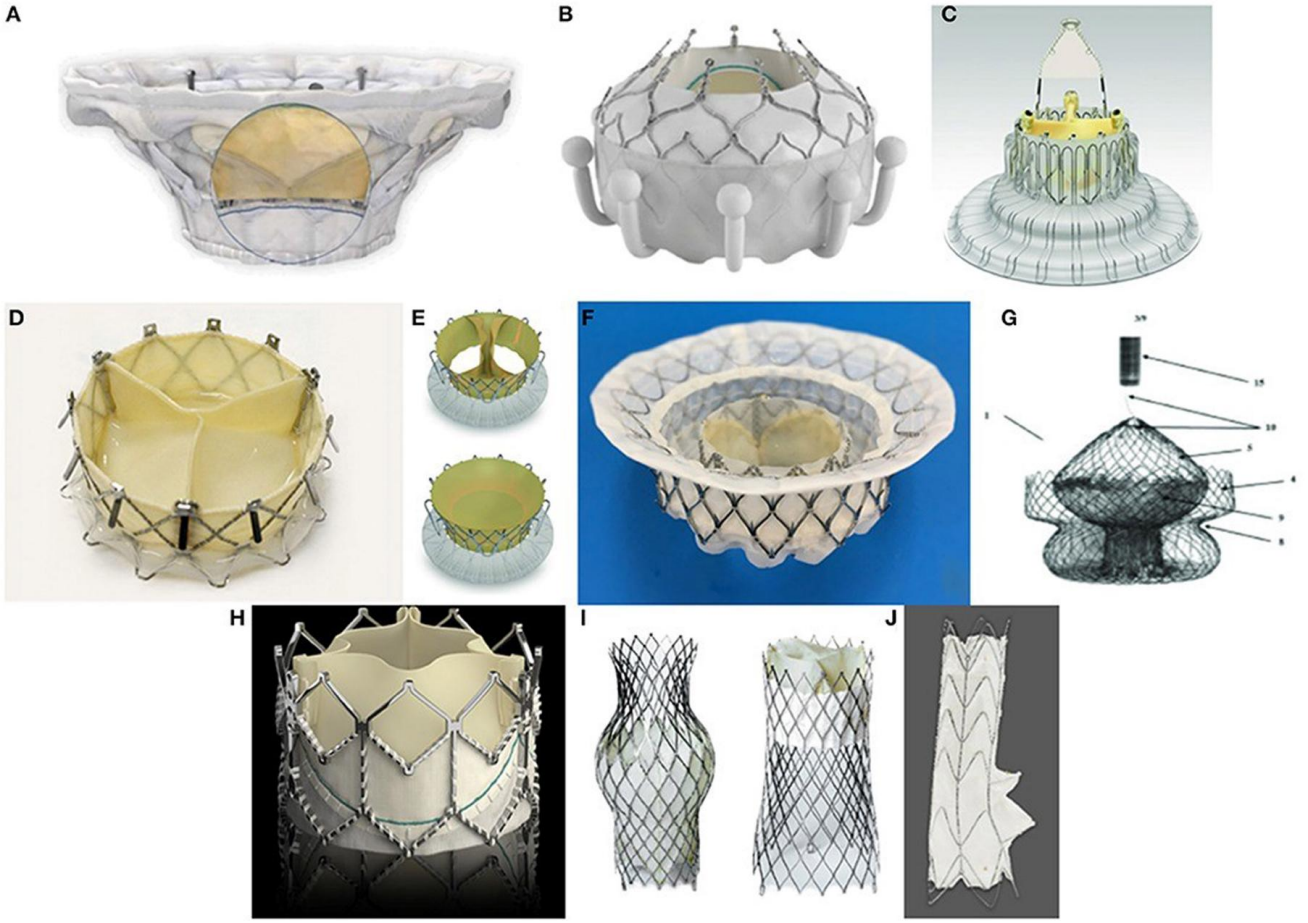
Representative TTVR products. (**A**) Cardiovalve, (**B**) evoque TV replacement system, (**C**) Lux-Valve, (**D**) the NaviGate THV, (**E**) the trisol valve, trisol medical, (**F**) intrepid, and (**G**) the TRiCares, are representative orthotopic TTVR devices under research. (**H**) The sapien line of balloon-expandible valves, (**I**) TricValve transcatheter bicaval valves, and (**J**) the TriCento bioprosthesis are representative heterotopic TTVR devices under research. Adapted from Ref. [5] with permission of Frontiers, © 2021.

Many patients with TR do not benefit from TTVr due to valve anatomy or unclear/poor image of transesophageal echocardiography [12]. Ultrasonic examination is limited by the structure and density of internal organs and tissues in the human body. The imaging quality and resolution of the TR site are not high [12]. The success rate of edge-to-edge leaflet repair significantly decreases when the effective regurgitant orifice area exceeds 0.7 cm^2^, the commissural fissure exceeds 6.5 mm, there is severe leaflet traction, or there is non-central/anterior septal commissural regurgitation [13]. In this case, TTVR may offer an alternative to break through the limitations of TTVr. TTVR can be broadly divided into orthotopic TTVR and heterotopic caval valve implantation (CAVI) [14]. Orthotopic TTVR deploys the valve at the TV annulus, while CAVI, first proposed by Lauten, implants the valve into the superior vena cava and/or inferior vena cava to prevent regurgitation volume to the atrium from backflowing to the vena cava [15]. Most TTVR devices are still in preclinical or early clinical trial stages (Figure 3), except Edwards Life Sciences’ EVOQUE tricuspid valve replacement system, which has received the CE Marking and FDA approval. The TTVR stent can be made of nickel-titanium alloy, cobalt-chromium alloy or stainless steel. The valve leaflets are generally made of animal-derived materials such as bovine pericardium [5, 16–18]. The sealing skirt that prevents paravalvular leakage is often made of animal-derived materials or polyethylene terephthalate (PET).

## Regulatory challenges

Despite being a highly promising technology, TTVR lacks medical consensus and guidance in the screening and selection of potential benefit groups and optimal intervention timing. Manufacturers have made varied attempts to enhance the effective anchoring of the intervention, reduce postoperative conduction block and thrombosis, and enhance long-term durability. This has led to large differences in TTVr/TTVR product design and architecture. It is difficult for regulatory agencies to rely on traditional approaches to review and evaluate the safety and efficacy of such diverse products. It is paramount to distinguish between risks inherent in product design and those occurring in clinical use caused by the lack of clinical guidelines. Understanding the nature of different risks and their interactions contributes to a more reasonable evaluation of the risk-benefit ratio.

### Risks inherent in product design and material composition

The anatomy of the TV is more complex than the mitral valve, with the former characterized by larger annulus and valve areas, greater individual variability and more fragile annular tissue structure [19]. The valve leaflets and chordae tendineae are also thinner, with irregular and easily expandable annular shapes. The extent of TR is easily affected by volume load. Due to the lack of valve and annular calcification and the complex adjacent structure, it is easy to cause damage to surrounding tissues, such as coronary artery damage due to proximity to the right coronary artery. Therefore, the structural design requirements of the device are high. For instance, it is required that the device should remain coaxial with the TV annulus after multiple extreme bends during device delivery, and the implant be effectively anchored without damaging surrounding tissues. Material innovation is conducive to the optimization of structural design, thereby enabling better performance. On the flip side, however, advanced materials also introduce a different set of risks and challenges. Unlike transcatheter aortic valve replacement devices, existing regulatory tools, technical evaluation methods and standards are not fully equipped for the evaluation of TTVr/TTVR device safety and efficacy. Alternative approaches are required to assess the material durability/fatigue performance, fluid mechanical properties and biological risks such as thrombosis, given the greater diversity of innovative structural design.

### Risks from clinical use

There are many causes of secondary TR [20]. Even patients with the same cause may have different TV anatomy, such as excessive spacing between TV leaflets, excessive valve annulus or the presence of pacemakers or defibrillator electrodes. Therefore, no single device may solve all the problems of different patients. The prognosis of the same patient using different devices may be different. Clinical judgment in selecting the right device for the right patient helps improve patient outcomes. Additionally, patients in the later stage of the disease course and with impaired right ventricular function may not benefit as much as those with intact function [21]. The results of TRILUMINATE Pivotal showed that there was no significant advantage for the device group in all-cause mortality and rehospitalization due to heart failure compared to the drug group [22]. However, the device group had much-improved quality-of-life scores. It seems that selecting patients in the early stage of disease progression produces higher clinical value [23]. The stage of TR disease can be considered as a factor in assessing the benefits to patients.

In summary, due to a lack of consistent clinical guidelines on if, when and how to perform TTVI therapies in TR patients, risks from clinical use will greatly interfere with the risk-benefit ratio of the devices for regulatory agencies.

## Regulatory science

Before medical device products are approved for market, regulatory agencies require a life-cycle evaluation to prove that the benefit of the product clearly outweighs the risk [7]. With traditional methods, regulatory agencies face challenges in evaluating the safety and efficacy of TTVr/TTVR devices given the uncertainty of clinical use and the diversity of innovative structural design. This section discusses the current state and future potential of regulatory scientific progress on TTVI products. The application of regulatory science is expected to promote the development and translation of TTVI products.

### Innovative approaches

Great importance is attached to innovative medical products around the world now. The FDA has established dedicated review and approval pathways for innovative medical devices to speed up access. For instance, Center for Devices and Radiological Health (CDRH) issues updated Final Guidance on Breakthrough Devices Program [24]. Edwards’ EVOQUE, the first FDA-approved TTVR product, was available through the Breakthrough Pathway [25].

In recent years, Chinese regulatory agencies issues policies and regulations to encourage the translation of innovative medical devices. For instance, in 2018, the Special Approval Procedures for Innovative Medical Devices was issued to formally establish a new pathway for innovative medical devices [26]. Medical Device Master Files were instituted for the raw materials of medical devices, integrating regulatory requirements for material technology and confidentiality disclosure [27]. Biomaterials Innovation and Cooperation Platform was built to help solve regulatory and technical bottlenecks in the translation of key biomaterials through the Leaders and Champions Program [28]. Regulatory science promoted the evaluation of the safety, efficacy and performance of innovative biomaterial medical devices and the translation of biomaterials [29, 30]. These measures have contributed to the rapid development of innovative medical devices in China.

TTVI products are distinguished from other types of innovative devices. Firstly, due to the lack of consistent clinical consensus or guidelines, the uncertainty of clinical use has a greater impact on the evaluation of product safety and efficacy. Secondly, due to the structural design diversity, the applicability of existing evaluation methods is yet to be confirmed. If the technical evaluation issues are to be resolved during the application for marketing approval, it will greatly increase the research and development (R&D) risks for innovators. The pre-review path proposed by the National Medical Product Administration (NMPA) of China moves upward the time for solving these technical evaluation issues. For a sponsor, the clinical benefits of products could be designed, validated and confirmed in the stage of R&D. This measure can help address these regulatory bottlenecks. Currently, TTVI products have been included in the key pre-review path program of NMPA.

### Computational modeling and simulations

Computational modeling and simulation (CM&S) is the application of computational modeling science and simulation technology to evaluate medical products [31–33]. As an excellent complement to traditional bench testing, it can evaluate the impact of non-circular configurations on transcatheter bioprosthetic heart valves [34–36]. It also helps analyze the causes of device damage, collects valve performance information that is difficult to obtain on the bench and effectively reduces wait time and economic costs [37, 38]. This technology is of great significance for optimizing the structural design of TTVI devices and improving performance such as fluid mechanics and valve durability.

**Figure 4.**
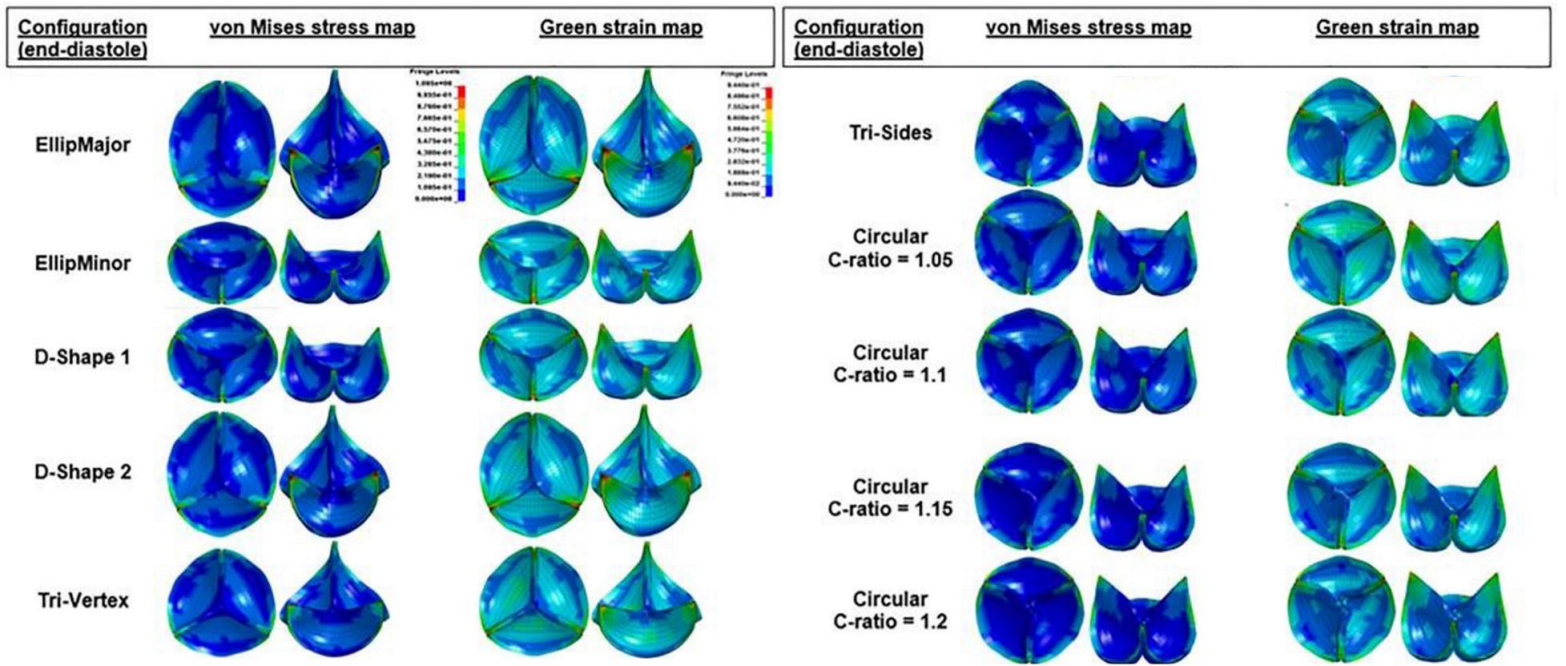
Stress and green strain distributions of the area-reduced valves at the end of diastole in different deformation configurations (top and 45° tilted views). The minimum and maximum of the fringe levels were set the same for all the stress or strain distributions for the sake of comparison. Sagging and stretched leaflets are also observed in the elliptical and D-shaped configurations. Pin-wheeling observed in a circular configuration with C-ratio = 1.2. Modified from Ref. [42] with permission of the American Society of Medical Engineers, © 2020.

The FDA has published a benchmark dataset for validating computational fluid dynamics (CFD) simulations of blood flow through generalized medical device geometries, providing validation data obtained from bench experiments within generic and simplified (i) nozzle and (ii) blood pump geometries [39]. The pressure and velocity data can be used for early-stage validation of their CFD model/software [40]. CM&S of clinically relevant bio-valve performance indicators can provide validation evidence for transcatheter heart valve (THV) motion, stress and strain, long-term deformability, valve morphology and calcification displacement, paravalvular leakage and postoperative hemodynamics [36–43]. (See Figure 4 for the stress and strain modeling and simulation as examples.)

### Developing accelerated wear testing methods for transcatheter heart valves

Clinical experience has shown that one of the major mechanisms of valve failure is non-calcific structural degeneration caused by cyclic leaflet bending and fatigue, leading to leaflet abrasions and tears [44–46]. Patterns of heart valve leaflet damage differ depending on the leaflet deformation. Free-edge tears and commissure tears are the primary causes of valve hydrodynamic incompetence. The observed pattern of leaflet damage is consistent with clinical evidence of fatigue-induced pericardial leaflet tears [47–49]. Sagging leaflets are more susceptible to free-edge tears than stretched or undistorted leaflets.

**Figure 5.**
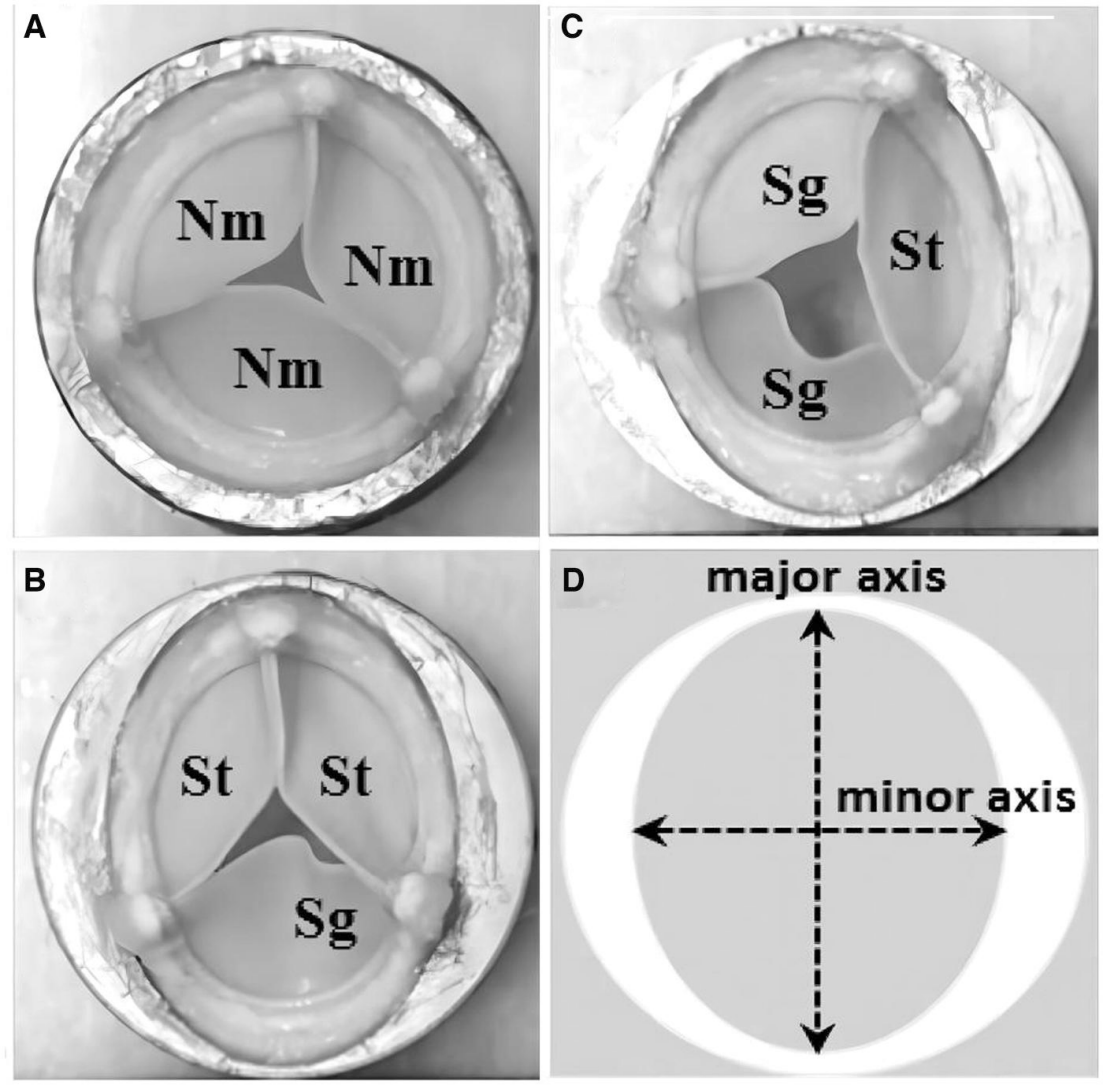
Representative valves in (**A**) the circular configuration with nominal (Nm, neither undistorted, sagging nor stretched) leaflets, (**B**) the EllipMajor configuration with two stretched (St) and one sagging (Sg) leaflets, and (**C**) the EllipMinor configuration with one stretched (St) and two sagging (Sg) leaflets. (**D**) Schematic showing the major and minor axes of the elliptical metallic adapter ring. Modified from Ref. [50] with the permission of Springer Link, © 2018.

There is a lack of mature testing methods for TTVI devices. For instance, there are no specific performance requirements and operability standards in the guidance. Sritharan et al. [50] provided an *in vitro* accelerated wear testing method. Figure 5 shows representative samples of each of the three valve configurations used for wear testing [50]. The valve was sutured to a metal adapter ring using fishing line (STREN Original) to simulate clinical procedures, and silicone (Silastic Type a, Dow Corning Corp, MI) was used around the valve to minimize perivalvular leakage.

### Evidence-based research

Evidence-based research (EBR) is research using methods such as systematic review and meta-analysis developed by evidence-based medicine [51–53]. EBR leverages accumulated evidence to identify the risks and benefits of medical products and offers a new method for evaluating the safety and efficacy of medical products [51–56]. At present, more evidence needs to be generated and collected on the feasibility, indications, patient selection, long-term performance and risks, and other issues related to the safety and efficacy of TTVr/TTVR products.

EBR in animal studies can provide scientific reference for clinical research design and long-term safety and efficacy evaluation of artificial valve products. A systematic review and meta-analysis of large animal studies may identify the risk of calcification in tissue-engineered heart valves. Systematic reviews and meta-analyses of prior evidence on catheter mitral valves may be useful in assessing post-TTVI mortality and TR [57, 58].

EBR in clinical studies can effectively reduce the uncontrollable risk in the clinical use of TTVI products, and help screen and select suitable patients and indications. For instance, EBR may be used to evaluate the efficacy and safety of isolated TV surgery in TR treatment [59], and those of concurrent TV repair in patients undergoing mitral valve surgery [60]. At present, EBR has begun in the TAVR field. Real-world data analysis can supplement the clinical evidence of potential benefits and risks of TTVI products, and long-term safety and efficacy, as well as indication optimization of such products [61, 62].

## Conclusion

Advances in clinical needs and emerging biomaterials and medical devices have promoted the development and translation of innovative TTVI devices. Accordingly, new risks must be considered, including the risks of product structure design and material composition and those from clinical use. These developments have created new challenges for regulatory agencies. Regulatory science will assist in the supervision of the entire life cycle of such products. At present, promising regulatory science areas for the safety and efficacy evaluation of TTVI products include new approaches for innovative devices, pre-review path, computer modeling and simulation, accelerated wear testing methods of THV and EBR. The results of regulatory science will be beneficial to the innovative development of TTVI devices.
